# VEGF121 como Mediador de Efeitos Cardioprotetores Pós-Hipóxia via CaSR e via da Protease Dependente de Mitocôndria

**DOI:** 10.36660/abc.20190902

**Published:** 2021-09-01

**Authors:** Yan Zhang, Wei-hua Yin, Fan Yang, Yun-qiang An, Wei Zhou, Hui Yu, Hong Xie, Yan-ling Zhang, Yue Zhu, Xiang-chun Shen, Ruiqing Tian

**Affiliations:** 1 Hospital of Guizhou Medical University Guiyang China Hospital of Guizhou Medical University, Guiyang - China; 2 Chinese Academy of Medical Sciences Beijing China Chinese Academy of Medical Sciences and Peking Union Medical College, Beijing - China; 3 Hospital of Guiyang Guiyang China The First People's Hospital of Guiyang, Guiyang – China

**Keywords:** Fator A de Crescimento do Endotélio Vascular, Receptores de Detecção de Cálcio, Doenças Cardiovasculares

## Abstract

**Fundamento:**

A doença cardiovascular é a principal causa de morte em todo o mundo. A apoptose mediada por hipóxia em cardiomiócitos é uma das principais causas de distúrbios cardiovasculares. O tratamento com a proteína do fator de crescimento endotelial vascular (VEGF, do inglês *vascular endothelial growth factor*) foi testado, mas as dificuldades operacionais limitaram seu uso. Entretanto, com os avanços da terapia gênica, aumentou o interesse na terapia gênica baseada no VEGF em doenças cardiovasculares. No entanto, o mecanismo preciso pelo qual a reposição de VEGF resgata os danos pós-hipóxia em cardiomiócitos não é conhecido.

**Objetivos:**

Investigar o efeito da expressão de VEGF121 pós-hipóxia utilizando cardiomiócitos de ratos neonatos.

**Métodos:**

Cardiomiócitos isolados de ratos neonatos foram utilizados para estabelecer um modelo *in vitro* de lesão cardíaca induzida por hipóxia. O efeito da superexpressão de VEGF, isolado ou em conjunto com inibidores de moléculas pequenas que têm como alvo os canais de cálcio, receptores sensíveis ao cálcio (CaSR, do inglês *calcium-sensitive receptors*) e calpaína, no crescimento e proliferação celular em lesão de cardiomiócitos induzidos por hipóxia, foram determinados com ensaio de MTT, coloração TUNEL, coloração com Anexina V/PI, lactato desidrogenase e atividade da caspase. Para análise estatística, um valor de p<0,05 foi considerado significativo.

**Resultados:**

Verificou-se que o efeito do VEGF121 foi mediado por CaSR e calpaína, mas não foi dependente dos canais de cálcio.

**Conclusões:**

Nossos resultados, mesmo em um ambiente *in vitro*, estabelecem as bases para uma validação futura e testes pré-clínicos da terapia gênica baseada em VEGF em doenças cardiovasculares.

## Introdução

A doença cardiovascular é a principal causa de morte em todo o mundo. O principal mecanismo de doença cardiovascular causada por hipóxia-isquemia é a apoptose de cardiomiócitos, a proliferação de fibroblastos e dano às células endoteliais vasculares, levando à remodelação vascular e disfunção cardíaca.^1^^,^^2^

Embora os tratamentos atuais, como angioplastia coronária e cirurgia de revascularização do miocárdio tenham melhorado significativamente o prognóstico em pacientes com doença cardiovascular e prolongado a vida, a taxa de mortalidade permanece alta.^3^ Nos últimos anos, com o rápido desenvolvimento da tecnologia de recombinação gênica, a terapia genética potencial para cardiomiopatia isquêmica tornou-se uma área de foco importante.^4^^–^^8^

A apoptose é um mecanismo importante para a deterioração da doença cardíaca após a hipóxia.^9^ A via de ativação da apoptose exógena estimula o receptor Fas através de fatores externos, como a hipóxia, e o complexo de morte na membrana celular é ativado, causando a apoptose. Na via de ativação endógena ou na via de apoptose mediada pela mitocôndria, o fator indutor de apoptose (AIF, do inglês *apoptosis-inducing factor*) e o citocromo c (Cyt-c) são liberados após a ativação do sinal do poro de transição de permeabilidade (PTP) mitocondrial, a proteína pró-apoptótica Bid é clivada para uma forma truncada chamada tBid, que juntos ativam a apoptose induzida pela caspase.^10^

A apoptose miocárdica está intimamente relacionada à sobrecarga de cálcio e acredita-se que esta sobrecarga esteja intimamente relacionada à via de apoptose mediada pela mitocôndria.^10^ O receptor sensível ao cálcio (CaSR), como um membro da família do receptor C acoplado à proteína G, mantém o Ca2+ e a homeostase de outros íons metálicos, regula o Ca2+ extracelular e atua como um inibidor e agonista no miocárdio.^11^ A calpaína é uma proteína apoptótica dependente da ativação da via mitocondrial de influxo de Ca2+. Após a ativação, ela pode induzir a ativação final da caspase-3, levando à apoptose dos cardiomiócitos.^12^

Foi demonstrado que os cardiomiócitos expressam receptores VEGF;^13^ a ligação do VEGF a esses receptores ativou as proteínas quinases ativadas por mitógenos.^14^ O VEGF também demonstrou promover a reentrada de cardiomiócitos no ciclo celular, levando ao aumento da divisão celular no coração. Este último processo, se não controlado, pode resultar em hipertrofia cardíaca.^15^ Utilizando um modelo de isquemia miocárdica crônica em porco, foi demonstrado que o VEGF também pode induzir a cariocinese de cardiomiócitos.^16^ Isso estabelece um papel importante e frequentemente negligenciado do VEGF na homeostase dos cardiomiócitos.

A terapia com proteína do fator de crescimento endotelial vascular (VEGF) tem sido utilizada para tratar doenças cardiovasculares. Entretanto, seu uso tem sido limitado devido ao alto custo da terapia com proteína de VEGF e a necessidade de repetidas administrações do medicamento.^17^ No entanto, com os avanços na terapia gênica, a pesquisa em terapia gênica com VEGF está sendo ativamente considerada. Todavia, o mecanismo através do qual a reposição do VEGF ocorre ainda precisa ser determinado. Aqui, utilizamos a transfecção mediada por lipossoma de VEGF121 em cardiomiócitos de cultura primária submetidos à hipóxia para investigar os efeitos *downstream* da reposição de VEGF em cardiomiócitos.

## Métodos

### Isolamento de cardiomiócito primário de rato

Este estudo foi aprovado pelo *Institutional Animal Care and Use Committee of The Affiliated Hospital of Guizhou Medical University* (Certificado de Aprovação nº SCXK (Qian) 2002-0001). Os cardiomiócitos foram isolados de ratos da linhagem Sprague-Dawley neonatos de 0-3 dias de idade, como descrito anteriormente.^14^

### Cultura de células primárias e agrupamento

Os cardiomiócitos foram contados e cultivados em placas de cultura de 6 cm revestidas com gelatina (1x10^7^ células/placa) em DMEM de alta glicose sem soro de bezerro. Havia 6 grupos experimentais – Grupo 1 (grupo controle negativo); Grupo 2 (grupo modelo de hipóxia); Grupo 3 (grupo modelo de hipóxia transfectado com VEGF121); Grupo 4 (igual ao Grupo 3, mas com inibidor do canal de cálcio CdCl2); Grupo 5 (igual ao Grupo 3, mas com inibidor do receptor CaSR NPS2390); Grupo 6 (igual ao Grupo 3, mas com inibidor da calpaína, calpeptina).

Para os Grupos 2-6, as células foram tratadas com solução tampão de isquemia (NaCl 137 mM, KCl 15,8 mM, MgCl2 0,49 mM, CaCl*2* 0,9 mM, HEPES 4 mM, 2-desoxiglicose 10 mM, lactato de sódio 20 mM e ditionito de sódio 1 mM, pH 6,5)^18^^,^^19^ por 2h. Para os Grupos 3-6, as células foram então transfectadas com pcDNA3.1(+)/VEGF121 utilizando Lipofectamina 2000 (Thermo Fisher Scientific), de acordo com as diretrizes do fabricante, e em seguida incubadas em DMEM contendo 8% de soro fetal bovino isolado (Grupo 3) ou com os respectivos inibidores (Grupos 4-6). Vinte e quatro horas após a transfecção, o sobrenadante de cardiomiócitos foi removido para ser utilizado.

### Ensaio MTT

O ensaio MTT (Sigma Millipore, EUA) foi utilizado para detectar o crescimento dos cardiomiócitos nos diferentes grupos experimentais, como descrito anteriormente.^20^ A absorvância foi medida a 570 nm.

### Atividade intracelular de lactato desidrogenase (LDH) e taxa de extravasamento de LDH

A atividade de LDH foi medida utilizando-se o kit Lactate Dehydrogenase Activity Assay (Sigma Millipore, EUA) de acordo com as instruções do fabricante. A taxa de extravasamento externa foi calculada como = [LDH do meio de cultura/(LDH do meio de cultura + LDH das células)] × 100%.

### Ensaio TUNEL

Para o ensaio TUNEL, os cardiomiócitos foram cultivados em placas de 24 poços (1x10^4^ células/poço) por 24h e fixados em paraformaldeído 4% por 15 minutos. Após duas lavagens com PBS, os cardiomiócitos foram incubados com solução de reação de desoxinucleotidil transferase terminal (TdT) por 2h a 37°C. As células coradas foram contadas utilizando microscópio fluorescente. A taxa de apoptose foi calculada de acordo com a fórmula: taxa de apoptose (%) = [número de células apoptóticas/número total de células] × 100%.

### Coloração com anexina V/PI

As células transfectadas foram desalojadas através de tratamento com Accutase (BD Biosciences, EUA) durante 2 minutos à temperatura ambiente, lavadas rapidamente em PBS e depois coradas com Anexina V-FITC e iodeto de propídio (PI; BD Biosciences, San Jose, CA, EUA). A análise de citometria de fluxo (Cytomics FC500; Beckman Coulter, Miami, FL, EUA) foi utilizada para medir as células apoptóticas. Os eventos de aquisição foram padronizados utilizando células de controle. A anexina V^+^PI^–^ e a anexina V^+^PI^+^ foram consideradas as células apoptóticas precoces e tardias, respectivamente.

### Análise da atividade da caspase-3

A atividade da caspase-3 foi medida utilizando um kit comercial de acordo com os protocolos do fabricante (APOPCYTO Caspase-3 Colorimetric Assay Kit; Medical and Biological Laboratories, Japão) como descrito em um estudo anterior.^21^

### Análise de Western blot

Ao final dos pontos de tempo experimentais, as células foram lavadas com PBS gelado e, em seguida, lisadas com solução-tampão RIPA. O lisado foi centrifugado a 12.000 g por 3 minutos e a concentração de proteína no sobrenadante foi quantificada através do kit BSA (Thermo Fisher, EUA). Vinte microgramas de proteína de cada amostra foi resolvida em SDS-PAGE e, em seguida, processada para *immunoblotting* (Western blot) utilizando metodologias de rotina.^14^ O *blot* recebeu sondas com anticorpos contra VEGF, CaSR, calpaína, AIF, a proteína pró-apoptótica Bid (tBid) truncada e β-actina (todos os anticorpos foram obtidos da Abcam, EUA). O mesmo blot foi removido e testado para diferentes anticorpos. A β-actina foi utilizada como controle de carregamento. A análise de densitometria foi realizada com o software NIH Image J.

### Análise estatística

A análise estatística foi realizada utilizando o software SPSS 20.0 (IBM Corporation, NY, EUA). Todos os dados foram apresentados como média ± desvio padrão (DP). Dado o pequeno tamanho da amostra, o teste de normalidade foi realizado com o teste de Kolmogorov-Smirnov. O teste *t* de Student não pareado foi utilizado para comparar as médias de dois grupos, enquanto as diferenças entre os vários grupos foram analisadas utilizando ANOVA de uma via. O tamanho da amostra não foi determinado por uma metodologia definida, mas foi amplamente estabelecido por conveniência; entretanto, cada experimento teve pelo menos três réplicas biológicas e técnicas independentes. Um valor de p <0,05 foi considerado estatisticamente significativo.

## Resultados

### A superexpressão de VEGF pode resgatar o crescimento celular de cardiomiócitos submetidos à hipóxia

**Figura 1:**
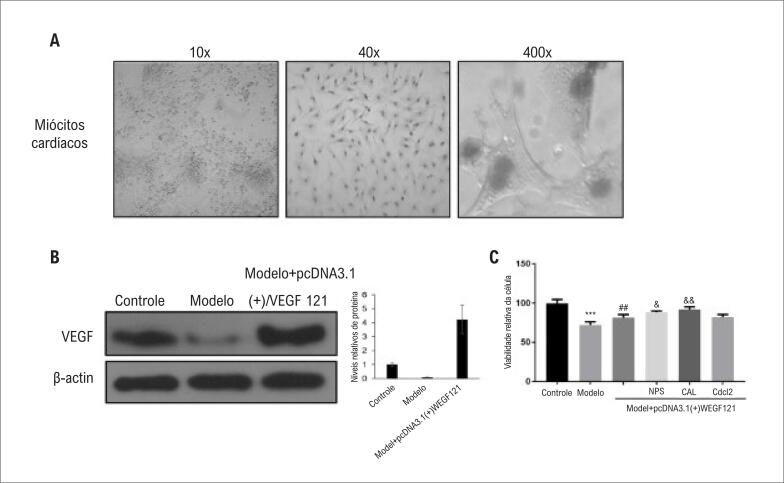
Modelo de hipóxia de cardiomiócitos de rato e determinação do crescimento de células de cardiomiócitos em diferentes condições experimentais. (A) Morfologia de miócitos cardíacos de rato neonatos normais após 3 dias (diferentes ampliações de 10x, 40x e 400x são mostradas). (B) Análise de Western blot de VEGF em miócitos cardíacos de rato neonato. O gráfico mostra a expressão relativa normalizada para β-actina. *P<0,05; n=6. (C) Ensaio de proliferação de MTT após tratamentos indicados (por 5h) em miócitos cardíacos de rato neonato. Os dados são representativos de 6 experimentos repetidos diferentes. ***P<0,001 vs. grupo controle; ^##^*P<0,01 vs. grupo modelo;* ^&^ *P<0,05*, ^&&^ *P <0,01 vs. grupo Modelo + pcDNA3.1 (+) / VEGF121*.

A contagem de células e as características morfológicas (Figura 1A) de cardiomiócitos cultivados em cultura primária do Grupo 1 no dia 3, confirmaram que os cardiomiócitos isolados estavam proliferando e crescendo de forma robusta *in vitro*. A coloração com azul de tripano foi utilizada para determinar a morte celular relativa após 3 dias de cultura, e mais de 95% dos cardiomiócitos eram viáveis. Em seguida, foi determinado o efeito da hipóxia (solução tampão de isquemia) na expressão da proteína de VEGF em cardiomiócitos (Grupo B). A cultura na solução tampão de isquemia diminuiu a expressão da proteína de VEGF (Figura 1B), indicando o estabelecimento bem-sucedido do sistema do modelo *in vitro*. A técnica de *immunoblotting* no Grupo C (grupo modelo de hipóxia transfectado com VEGF121) confirmou a superexpressão bem-sucedida de VEGF (Figura 1B).

Em seguida, o ensaio MTT foi utilizado para determinar o crescimento celular relativo nos diferentes grupos experimentais em comparação com o Grupo 1 (controle negativo). A hipóxia inibiu significativamente o crescimento celular no Grupo 2 (grupo modelo) em comparação com o Grupo 1 (Figura 1C; p <0,001). Em seguida, o efeito da superexpressão de VEGF121 foi testado isoladamente ou em combinação com o inibidor do canal de Ca2+ CdCl2, o inibidor de CaSR NPS2390 e o inibidor de calpaína, a calpeptina. A superexpressão de VEGF121 aumentou significativamente o crescimento celular nos cardiomiócitos expostos à hipóxia. A adição adicional de NPS2390 e calpeptina, mas não de CdCl2, resultou em um efeito sinérgico, aumentando ainda mais o crescimento dos cardiomiócitos (Figura 1C).

### CdCl2CdCl2 Detecção de apoptose de cardiomiócitos

**Figura 2:**
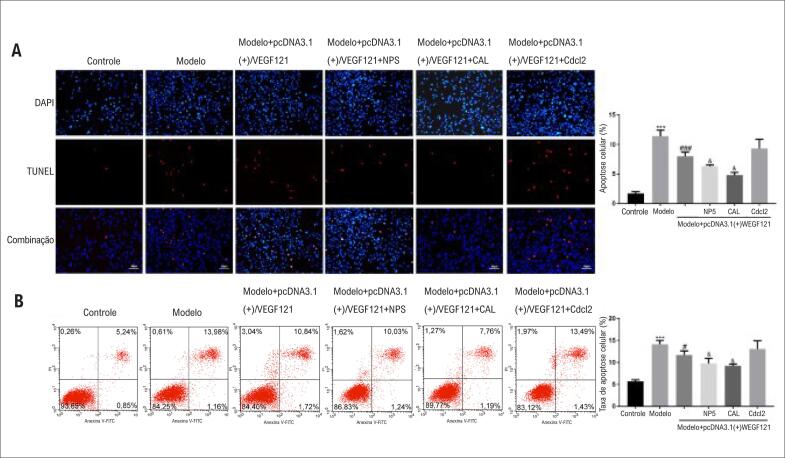
*O VEGF pode resgatar morte celular induzida por hipóxia em cardiomiócitos. Os grupos experimentais foram - Grupo 1 (grupo controle negativo); Grupo 2 (grupo modelo de hipóxia); Grupo 3 (grupo modelo de hipóxia transfectado com VEGF121); Grupo 4 (igual ao Grupo 3, mas com inibidor do canal de cálcio CdCl2); Grupo 5 (igual ao Grupo 3, mas com inibidor do receptor CaSR NPS2390); Grupo 6 (igual ao Grupo 3, mas com inibidor da calpaína Calpeptina). (A) Coloração TUNEL de miócitos cardíacos após diferentes tratamentos. O gráfico mostra a quantificação das imagens. (B) Coloração com anexina V/PI de miócitos cardíacos após diferentes tratamentos. O gráfico mostra a quantificação dos dados de citometria de fluxo.* ***p<0,001 vs. grupo controle; ###*p <0,01 vs. grupo modelo;* &*p<0,05 vs. grupo modelo + pcDNA3.1(+)/VEGF121; n = 10 em A e n = 3 em B*.

Considerando que a superexpressão de VEGF demonstrou estar aumentando a proliferação de células de cardiomiócitos, decidimos investigar se a superexpressão de VEGF também estava atenuando a morte apoptótica induzida por hipóxia nos cardiomiócitos. A apoptose foi detectada pela coloração de TUNEL e Anexina V/PI. Em comparação com o Grupo 1 controle negativo, a hipóxia induziu morte celular significativa por apoptose (Figura 2A, B). A superexpressão de VEGF121 atenuou significativamente a morte celular induzida por hipóxia (Figura 2A, B). Tal como acontece com a proliferação celular, tanto o NPS2390 quanto a calpeptina apresentaram efeito sinérgico com a superexpressão de VEGF121 na atenuação da morte celular induzida por hipóxia, enquanto a adição de CdCl2 não teve nenhum efeito adicional (Figura 2A, B). Tomados em conjunto, esses resultados indicaram que a expressão de VEGF atenua a morte celular induzida por hipóxia via ativação do receptor CaSR e calpaína.

### Efeitos do VEGF no extravasamento de LDH e na atividade da caspase-3

**Figura 3:**
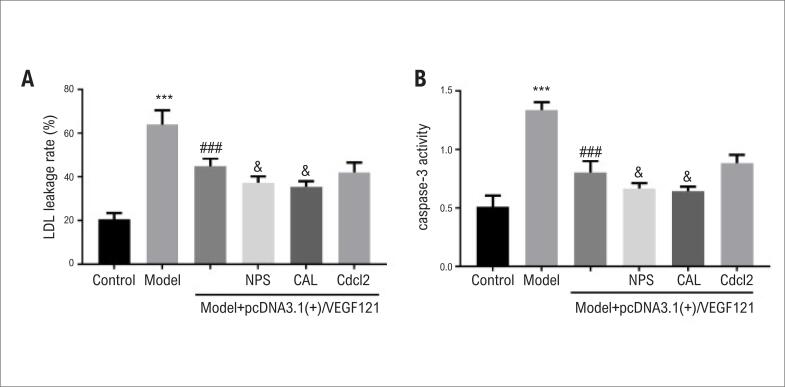
*O VEGF pode inibir o extravasamento de LDH e a atividade da Caspase-3 em cardiomiócitos tratados com hipóxia. Os grupos experimentais foram - Grupo 1 (grupo controle negativo); Grupo 2 (grupo modelo de hipóxia); Grupo 3 (grupo modelo de hipóxia transfectado com VEGF121); Grupo 4 (igual ao Grupo 3, mas com inibidor do canal de cálcio CdCl2); Grupo 5 (igual ao Grupo 3, mas com inibidor do receptor de CaSR NPS2390); Grupo 6 (igual ao Grupo 3, mas com inibidor da calpaína Calpeptina). São mostrados dados para o extravasamento de LDH (A) e atividade da caspase-3 (B) de cinco experimentos repetidos independentes.* ***p<0,001 vs. grupo controle; ^###^*p<0,05 vs. grupo modelo;* ^&^*p <0,05 vs. grupo modelo + pcDNA3.1(+)/VEGF121*.

Em seguida, investigamos se o efeito do VEGF na inibição da morte celular em cardiomiócitos estava sendo mediado pela diminuição do extravasamento de LDH e/ou diminuição da atividade da caspase-3. A atividade de LDH em cardiomiócitos foi medida e os resultados mostraram que a taxa de extravasamento de LDH em cardiomiócitos aumentou após a indução de hipóxia (p<0,001). A superexpressão de VEGF121 diminuiu significativamente a taxa de extravasamento de LDH (Figura 3A). Tanto o NPS2390 quanto a calpeptina apresentaram efeito sinérgico com a superexpressão de VEGF121 na redução da taxa de extravasamento de LDH, enquanto a adição de CdCl2 não mostrou nenhum efeito adicional (Figura 3A). Uma observação semelhante foi feita com a atividade da caspase-3 (Figura 3B).

### Impacto da hipóxia e VEGF na expressão de CaSR, Calpaína, AIF e tBid

**Figura 4:**
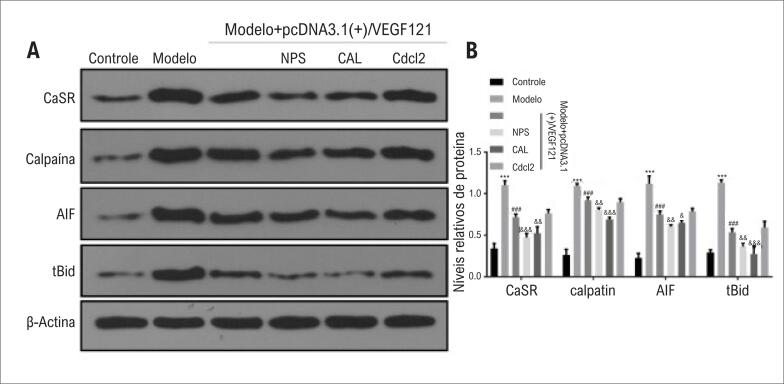
Impacto da hipóxia, VEGF e inibidores indicados na expressão da proteína CaSR, Calpaína, AIF e tBid. Os grupos experimentais foram - Grupo 1 (grupo controle negativo); Grupo 2 (grupo modelo de hipóxia); Grupo 3 (grupo modelo de hipóxia transfectado com VEGF121); Grupo 4 (igual ao Grupo 3, mas com inibidor do canal de cálcio CdCl2); Grupo 5 (igual ao Grupo 3, mas com inibidor do receptor CaSR NPS2390); Grupo 6 (igual ao Grupo 3, mas com inibidor da calpaína Calpeptina). (A, B) CaSR, Calpaína, AIF e tBid foram detectados pela análise de Western blotting. A Figura mostra os blots de 4 experimentos independentes. Os gráficos mostram a análise densitométrica dos blots dos quatro experimentos repetidos. ***p <0,001 vs. grupo controle; ^###^ *p<0,001 vs. grupo modelo;* ^&^ *p<0,05*, ^&&^ *p<0,01*, ^&&&^ *p <0,001 vs. grupo Modelo + pcDNA 3.1(+)/VEGF121*.

A hipóxia induziu significativamente a expressão das proteínas CaSR, calpaína, AIF e tBid (Figura 4; p<0,001). Os níveis de expressão de CaSR, calpaína, AIF e proteína tBid diminuíram após a transfecção de cardiomiócitos com pcDNA 3.1(+)/VEGF121, o que foi estatisticamente significativo em comparação com o grupo modelo de hipóxia (p<0,001). Após o tratamento dos cardiomiócitos com NPS2390 e Calpeptina, a expressão de tBid e AIF diminuiu em comparação com o grupo do Modelo + pcDNA 3.1(+)/VEGF121 (p<0,05). Após a intervenção com pcDNA 3.1(+)/VEGF121 em cardiomiócitos, a expressão de CaSR diminuiu, o que foi estatisticamente significativo em comparação com o grupo modelo (p<0,001). Novamente, o efeito sinérgico foi observado com NPS2390 e calpeptina, mas não com CdCl2.

## Discussão

Os principais mecanismos de doença cardiovascular causada por hipóxia-isquemia são a apoptose miocárdica, proliferação de fibroblastos e lesão de células endoteliais vasculares.^22^^–^^24^ O VEGF, um mitógeno específico de células endoteliais vasculares, representa uma família de proteínas secretadas, incluindo VEGF121, VEGF165, VEGF189 e VEGF206, dos quais o VEGF121 e o VEGF165 são os principais tratamentos para a cardiomiopatia isquêmica. Foi relatado que o VEGF121 só pode se ligar ao receptor VEGFR-2, e sua ligação específica ao VEGF pode atenuar a angiogênese, mas outros estudos também descobriram que sua capacidade de se ligar à heparina é enfraquecida, o que é mais propício ao ambiente anóxico. A injeção direta do plasmídeo VEGF121 no modelo de infarto do miocárdio de porco demonstrou melhorar o suprimento de sangue e aumentar o número de vasos colaterais ao mesmo tempo, destacando o potencial da terapia genética baseada em VEGF. No entanto, existem limitações de instabilidade, preço alto e falta de aplicação de altas doses na prática clínica. Ao mesmo tempo, estudos também descobriram que o produto do gene que codifica a proteína secretada tem um efeito parácrino, em comparação com a proteína da própria célula.^25^

Os cardiomiócitos são células terminalmente diferenciadas, cuja apoptose está intimamente relacionada à doença miocárdica. A caspase-3 é o mediador comum de apoptose em cardiomiócitos.^26^^,^^27^ A insuficiência cardíaca mostra diferentes taxas de apoptose de cardiomiócitos. Em experimentos com insuficiência cardíaca em animais causada por hipóxia-reperfusão, a taxa apoptótica de cardiomiócitos pode chegar a 14%, e a insuficiência cardíaca é causada por excesso de sobrecarga de pressão. A taxa de apoptose dos cardiomiócitos no modelo é de apenas 1%,^24^ o que sugere que o estudo da apoptose em modelo de hipóxia miocárdica é de grande valor.

A análise de *imunoblotting* confirmou a expressão bem-sucedida de VEGF121 em cardiomiócitos pós-transfecção no estudo atual. Alguns estudos também confirmaram que a quantificação^9^ de VEGF121 no sobrenadante celular pode confirmar a secreção e expressão de cardiomiócitos de um vetor de expressão eucariótico e tem um forte efeito proliferativo nas células endoteliais. A LDH atua como uma taxa de extravasamento extra para enzimas intracelulares e seu aumento é um marcador de dano celular. A taxa de extravasamento de LDH no grupo do modelo hipóxico foi alta e resgatada pelo VEGF121.

A ativação da caspase-3 na hipóxia foi inibida pelo VEGF121 e o inibidor de CaSR NPS2390, sugerindo que o receptor de calpaína e a calpaína estão intimamente relacionados ao mecanismo de apoptose de cardiomiócitos.^10^ Considrando que nossos achados sugeriram que o VEGF121 reverte a apoptose em cardiomiócitos pós-hipóxia e o inibidor de CaSR tem um efeito semelhante, supõe-se que o VEGF121 esteja mediando sua atividade ao inibir a ativação do CaSR.

Como as alterações dinâmicas dos níveis de cálcio intracelular são um dos mecanismos importantes da apoptose,^28^^,^^29^ as vias intracelulares que afetam os níveis de cálcio principalmente através dos canais de Ca2+ e da troca Na+/Ca2+, ativam as vias do receptor de Ca2+, como CaSR. Em outras vias,^30^ quando a hipóxia é estimulada, o influxo de Ca2+ intracelular aumenta, CaN e Bad são ativados, e a permeabilidade da membrana mitocondrial é significativamente aumentada, liberando Cyt-c para ativação citoplasmática de Caspase-3, promovendo assim dano aos cardiomiócitos. Uma vez que calpaína é dependente da ativação do influxo de Ca2+, a calpaína ativada pode clivar e ativar a Bid. Devido ao seu domínio BH3 especial, a tBid pode induzir a translocação de Bax/Bak, resultando em aumento da permeabilidade da membrana mitocondrial, e tBid também pode passar através dela. O domínio não-BH3 induz alterações estruturais da membrana mitocondrial, abre o PTP e ativa a calpaína para degradar proteínas inibitórias de CaN e, assim, ativando CaN, que promove a fosforilação de Bad, levando à apoptose.^31^^–^^33^

A Bid é única proteína da família Bcl-2 que pode ser clivada em uma pequena molécula Bid. Após sua ativação, ela promove a polimerização de Bak e Bax, ou se homopolimeriza, promovendo o remodelamento da membrana mitocondrial interna e a abertura dos mPTP (poros de transição de permeabilidade mitocondrial), resultando na liberação de Cyt-c.^34^^,^^35^ Nossos resultados mostraram que a expressão de tBid em cardiomiócitos hipóxicos estava significativamente aumentada. O nível de tBid diminuiu com a superexpressão de VEGF e diminuiu ainda mais sinergicamente com a adição de NPS2390 e calpeptina, mas não com a adição de CdCl2. Isso indicou que o íon cálcio e a calpaína causaram uma diminuição na atividade de Bid após a apoptose miocárdica causada pela hipóxia. O AIF está localizado na membrana mitocondrial interna. Quando a apoptose dos cardiomiócitos ocorre, a protease flavina, com atividade da NADH oxidase, é liberada da mitocôndria para o citoplasma e, em seguida, translocada para o núcleo, envolvida na condensação da cromatina e fragmentação do DNA.^36^ Neste experimento, o inibidor do receptor CaSR (NPS2390) e o inibidor da calpaína (calpeptina) foram adicionados para induzir uma diminuição nos níveis da proteína AIF, a qual foi estatisticamente significativa. Nossos resultados sugeriram que o VEGF121 está envolvido na apoptose dos cardiomiócitos após AIF. A liberação foi associada à ativação de CaSR, enquanto o grupo de antagonista da calpaína calpeptina e o grupo de intervenção pcDNA 3.1(+)/VEGF121 não foram estatisticamente significativos, sugerindo que não é exclusivamente dependente das vias *downstream* de apoptose induzida por protease dependente de cálcio.

Uma possível limitação de nosso estudo é que os experimentos foram realizados com cardiomiócitos neonatais; a relevância precisa ser investigada em cardiomiócitos isolados de ratos mais velhos e em modelos pré-clínicos *in vivo*.

## Conclusão

Foi demonstrado anteriormente que o VEGF pode tratar a isquemia miocárdica, aumentar a proliferação de células endoteliais vasculares, aumentar a circulação colateral miocárdica e a densidade capilar, melhorando assim o suprimento sanguíneo miocárdico na área isquêmica e melhorando a função cardíaca.^37^^–^^39^ Entretanto, danos às células endoteliais vasculares, hipertrofia de cardiomiócitos, apoptose de cardiomiócitos e proliferação de fibroblastos também são alterações fisiopatológicas importantes da miocardiopatia isquêmica. Neste estudo, com base nos grupos de intervenção, o VEGF121 afetou a proliferação dos cardiomiócitos, confirmando a atividade biológica dos plasmídeos eucariotos sobre os cardiomiócitos. Em conclusão, nossos resultados fornecem uma base para futuras abordagens de terapia genética baseada em VEGF em doenças cardiovasculares.
