# Supplementary material for: VEGF121 como Mediador de Efeitos Cardioprotetores Pós-Hipóxia via CaSR e via da Protease Dependente de Mitocôndria
Source: Arq Bras Cardiol. 2021 Sep 1;117(3):476–83. [Article in Portuguese] doi: 10.36660/abc.20190902 (PMC8462959; doi:10.36660/abc.20190902)
Supplement: Translation

# Translation: VEGF121 Mediates Post-Hypoxia Cardioprotective Effects Via CaSR and Mitochondria-Dependent Protease Pathway

**Authors:** Yan Zhang, Wei-hua Yin, Fan Yang, Yun-qiang An, Wei Zhou, Hui Yu, Hong Xie, Yan-ling Zhang, Yue Zhu, Xiang-chun Shen, Ruiqing Tian

**Affiliations:** 1 Guiyang China Hospital of Guizhou Medical University, Guiyang - China; 2 Beijing China Chinese Academy of Medical Sciences and Peking Union Medical College, Beijing - China; 3 Guiyang China The First People's Hospital of Guiyang, Guiyang – China

**Keywords:** Vascular Endothelial Growth Factor A, Calcium Sensing Receptors, Cardiovascular Disease

## Abstract

**Background:**

Cardiovascular disease is the major cause of death worldwide. Hypoxia-mediated apoptosis in cardiomyocytes is a major cause of cardiovascular disorders. Treatment with vascular endothelial growth factor (VEGF) protein has been tested but operational difficulties have limited its use. However, with the advancements of gene therapy, interest has risen in VEGF-based gene therapy in cardiovascular disorders. However, the precise mechanism by which VEGF replenishment rescues post-hypoxia damage in cardiomyocytes is not known.

**Objectives:**

To investigate the effect of post-hypoxia VEGF121 expression using neonatal rat cardiomyocytes.

**Methods:**

Cardiomyocytes isolated from neonatal rats were used to establish an in vitro model of hypoxia-induced cardiac injury. The effect of VEGF overexpression, alone or in combination with small-molecule inhibitors targeting calcium channel, calcium sensitive receptors (CaSR), and calpain on cell growth and proliferation on hypoxia-induced cardiomyocyte injury were determined using an MTT assay, TUNEL staining, Annexin V/PI staining, lactate dehydrogenase and caspase activity. For statistical analysis, a value of P<0.05 was considered to be significant.

**Results:**

The effect of VEGF121 was found to be mediated by CaSR and calpain but was not dependent on calcium channels.

**Conclusions:**

Our findings, even though using an in vitro setting, lay the foundation for future validation and pre-clinical testing of VEGF-based gene therapy in cardiovascular diseases.

## Introduction

Cardiovascular disease is the major cause of death worldwide. The main mechanism of cardiovascular disease caused by hypoxia-ischemia is cardiomyocyte apoptosis, fibroblast proliferation and vascular endothelial cell damage, leading to vascular remodeling and cardiac dysfunction.^1^^,^^2^ Although current treatments such as coronary angioplasty and coronary artery bypass grafting have significantly improved the prognosis in patients with cardiovascular disease and prolonged life, the mortality rate remains high.^3^ In recent years, with the rapid development of genetic recombination technology, potential gene therapy for ischemic cardiomyopathy has become an area of major focus.^4^^–^^8^

Apoptosis is an important mechanism for the deterioration of heart disease after hypoxia.^9^ The exogenous apoptosis activation pathway stimulates the Fas receptor through external factors such as hypoxia, and the death complex in the cell membrane is activated, causing apoptosis. In the endogenous activation pathway or the mitochondria-mediated apoptosis pathway, the apoptosis-inducing factor (AIF) and cytochrome c (Cyt-c) are released after the activation of mitochondrial permeability transition pore (PTP) signal, the pro-apoptotic protein Bid is cleaved into a truncated form called tBid, which together activate caspase-induced apoptosis.^10^

Myocardial apoptosis is closely related to calcium overload, and it is believed that calcium overload is closely related to the mitochondria-mediated apoptosis pathway.^10^ The calcium-sensing receptor (CaSR), as a member of the G-protein coupled receptor C-family, maintains Ca2+ and other metal ion homeostasis, regulates extracellular Ca2+, and acts as an inhibitor and agonist in the myocardium.^11^ Calpain is an apoptotic protein that is dependent on the activation of the mitochondrial pathway of Ca2+ influx. Upon activation, it can induce the final activation of caspase-3, leading to cardiomyocyte apoptosis.^12^

It has been shown that cardiomyocytes express VEGF receptors;^13^ the binding of VEGF to these receptors activate mitogen-activated protein kinases.^14^ VEGF has also been shown to promote the re-entrance of cardiomyocytes into the cell cycle, leading to an increase in cell division in the heart. The latter process, if not controlled, can end up in cardiac hypertrophy.^15^ Using a pig model of chronic myocardial ischemia, it has been shown that VEGF can also induce cardiomyocyte karyokinesis.^16^ This establishes an important and often neglected role of VEGF in cardiomyocyte homeostasis.

Vascular endothelial growth factor (VEGF) protein therapy has been used to treat cardiovascular disease. However, it has been limited due to the high cost of VEGF protein therapy and the need for repeated drug administration.^17^ However, with the advances made in gene therapy, VEGF gene therapy research is being actively considered. However, the mechanism through which replenishment of VEGF works remains to be determined. Here, we used liposome-mediated transfection of VEGF121 into primary cultured cardiomyocytes submitted to hypoxia to investigate the downstream effects of VEGF replenishment in cardiomyocytes.

## Methods

### Isolation of rat primary cardiomyocyte

This study was approved by the Institutional Animal Care and Use Committee of The Affiliated Hospital of Guizhou Medical University (Approval Certificate No. SCXK (Qian) 2002-0001). Cardiomyocytes were isolated from 0-3-day-old neonatal Sprague Dawley rats, as previously described.^14^

### Primary cell culture and grouping

The cardiomyocytes were counted and cultured in gelatin-coated 6-cm culture dishes (1x10^7^cells/dish) in high-glucose DMEM, without calf serum. There were 6 experimental groups - Group 1 (negative control group); Group 2 (hypoxia model group); Group 3 (hypoxia model group transfected with VEGF121); Group 4 (same as Group 3, but with calcium-channel inhibitor CdCl2); Group 5 (same as Group 3 but with CaSR receptor inhibitor NPS2390); Group 6 (same as Group 3 but with calpain inhibitor Calpeptin).

For Groups 2-6, the cells were treated with ischemia buffer (137 mM NaCl, 15.8 mM KCl, 0.49 mM MgCl_2_, 0.9 mM CaCl_2_, 4 mM HEPES, 10 mM 2-deoxyglucose, 20 mM sodium lactate and 1 mM sodium dithionite, pH 6.5)^18^^,^^19^ for 2 h. For Groups 3-6, cells were then transfected with pcDNA3.1(+)/VEGF121 using Lipofectamine 2000 (Thermo Fisher Scientific) according to the manufacturer's guidelines and then incubated in DMEM containing 8% fetal bovine serum alone (Group 3) or with the respective inhibitors (Groups 4-6). The 24-h post-transfection supernatant of the cardiomyocytes was removed for use.

### MTT assay

The MTT assay (Sigma Millipore, USA) was used to detect cardiomyocyte growth in the different experimental groups, as previously described.^20^ Absorbance was measured at 570 nm.

### Intracellular lactate dehydrogenase (LDH) activity and LDH leakage rate

The LDH activity was measured using the Lactate Dehydrogenase Activity Assay Kit (Sigma Millipore, USA) following the manufacturer's instructions. External leakage rate was calculated as = [culture medium LDH / (culture medium LDH + cell LDH)] × 100%.

### TUNEL assay

For the TUNEL assay, the cardiomyocytes were cultured in 24-well plates (1x10^4^ cells/well) for 24h and fixed in 4% paraformaldehyde for 15min. After two washes with PBS, the cardiomyocytes were incubated with Terminal deoxynucleotidyl transferase (TdT) reaction solution for 2h at 37°C. Stained cells were counted using a fluorescent microscope. The apoptosis rate was calculated according to the formula: apoptosis rate (%) = [number of apoptotic cells/total number of cells] ×100%.

### Annexin V/PI staining

The transfected cells were dislodged by treatment with Accutase (BD Biosciences, USA) for 2 minutes at room temperature, quickly washed in PBS and then stained with Annexin V-FITC and propidium iodide (PI; BD Biosciences, San Jose, CA, USA). Flow cytometry analysis (Cytomics FC500; Beckman Coulter, Miami, FL, USA) was used to measure the apoptotic cells. Acquisition events were standardized by using control cells. Annexin V^+^PI^–^ and Annexin V^+^PI^+^ were considered as the early and late apoptotic cells, respectively.

### Analysis of caspase-3 activity

Caspase-3 activity was measured using a commercial kit, according to the manufacturer's protocols (APOPCYTO Caspase-3 Colorimetric Assay Kit; Medical and Biological Laboratories, Japan) as described in a previous study.^21^

### Western blot analysis

At the end of the experimental time points, the cells were washed with ice-cold PBS and then lysed using RIPA buffer. The lysate was centrifuged at 12,000 g for 3 minutes and the protein concentration in the supernatant was quantified using a BSA kit (Thermo Fisher, USA). Twenty micrograms of protein of each sample was resolved by SDS-PAGE and then processed for immunoblotting, using routine methodologies.^14^ The blot was probed with antibodies against VEGF, CASR, calpain, AIF, the pro-apoptotic protein truncated Bid (tBid), and β-actin (all antibodies obtained from Abcam, USA). The same blot was stripped and probed for different antibodies. β-actin was used as a loading control. The densitometry analysis was performed using the NIH Image J software.

### Statistical analysis

The statistical analysis was performed using SPSS 20.0 (IBM Corporation, NY, USA). All data are shown as mean ± standard deviation (SD). Given the small sample size, the normality test was performed using the Kolmogorov-Smirnov test. The unpaired Student's t-test was used to compare the means of two groups, while the differences between multiple groups were analyzed using one-way ANOVA. The sample size was not determined by a set methodology but was largely dictated by convenience; however, every experiment had at least three independent biological and technical replicates. A value of p< 0.05 was considered as statistically significant.

## Results

### VEGF overexpression can rescue cell growth of cardiomyocytes submitted to hypoxia

Cell count and morphological characteristics (Figure 1A) of primary cultured cardiomyocytes from Group 1 on day 3 confirmed that the isolated cardiomyocytes were proliferating and growing robustly *in vitro*. Trypan blue staining was used to determine relative cell death after 3 days in culture and more than 95% of the cardiomyocytes were viable. Then, the effect of hypoxia (ischemia buffer) on VEGF protein expression in cardiomyocytes (Group B) was determined. The culture in the ischemia buffer decreased VEGF protein expression (Figure 1B), indicating successful establishment of the *in vitro* model system. Immunoblotting in Group C (hypoxia model group transfected with VEGF121) confirmed the successful overexpression of VEGF (Figure 1B).

**Figure 1:** Rat cardiomyocyte hypoxia model and determination of cardiomyocyte cell growth under different experimental conditions. (A) Morphology of normal neonatal rat cardiac myocytes after 3 days (different magnifications of 10x, 40x, and 400x are shown). (B) Western blot analysis of VEGF in neonatal rat cardiac myocytes. The graph shows the relative expression normalized to β-actin. *p<0.05; n=6. (C) the MTT proliferation assay after the indicated treatments (for 5h) in neonatal rat cardiac myocytes. Data is representative of 6 different experimental replicates. ***p <0.001 vs. control group; ^##^ *p<0.01 vs. Model group;* ^&^ *p<0.05*, ^&&^ *p<0.01 vs. Model + pcDNA3.1(+)/VEGF121 group*.

Subsequently, the MTT assay was used to determine relative cell growth in the different experimental groups when compared to Group 1 (negative control). Hypoxia significantly inhibited cell growth in Group 2 (model group) compared to Group 1 (Figure 1C; p<0.001). Next, the effect of VEGF121 overexpression was tested alone or in combination with Ca2+ channel inhibitor CdCl2, CaSR inhibitor NPS2390, and calpain inhibitor calpeptin. VEGF121 overexpression significantly increased cell growth in the cardiomyocytes exposed to hypoxia. Further addition of NPS2390 and calpeptin, but not CdCl2, resulted in a synergistic effect, further increasing cardiomyocyte growth (Figure 1C).

### Detection of cardiomyocyte apoptosis

Given that VEGF overexpression showed to be increasing cardiomyocyte cell proliferation, it was decided to investigate whether VEGF overexpression was also attenuating hypoxia-induced apoptotic death in the cardiomyocytes. Apoptosis was detected by TUNEL and Annexin V/PI staining. Compared to the negative control Group 1, hypoxia induced significant apoptotic cell death (Figure 2A, B). VEGF121 overexpression significantly attenuated hypoxia-induced cell death (Figure 2A, B). As with cell proliferation, both NPS2390 and calpeptin had a synergistic effect with VEGF121 overexpression in attenuating hypoxia-induced cell death, whereas the addition of CdCl2 did not have any additional effect (Figure 2A, B). Taken together, these results indicated that VEGF expression mitigates hypoxia-induced cell death via CaSR receptor activation and calpain.

**Figure 2:** *VEGF can rescue hypoxia-induced cell death in cardiomyocytes. Experimental groups were - Group 1 (negative control group); Group 2 (hypoxia model group); Group 3 (hypoxia model group transfected with VEGF121); Group 4 (same as Group 3 but with calcium channel inhibitor CdCl2); Group 5 (same as Group 3 but with CaSR receptor inhibitor NPS2390); Group 6 (same as Group 3 but with calpain inhibitor Calpeptin). (A) TUNEL staining of cardiac myocytes following different treatments. Graph shows quantification of images. (B) Annexin V/PI staining of cardiac myocytes following different treatments. Graph shows quantification of flow cytometry data.* ***p <0.001 vs. control group; ^###^ *p<0.01 vs. Model group;* ^&^ *p<0.05 vs. Model + pcDNA3.1(+)/VEGF121 group; n=10 in A and n=3 in B*.

### Effects of VEGF on the LDH release and caspase-3 activity

Next, it was investigated whether the effect of VEGF on cell death inhibition in cardiomyocytes was being mediated by decreasing LDH release and/or decreasing caspase-3 activity. LDH-activity in cardiomyocytes was measured, and the results showed that the LDH leakage rate in cardiomyocytes was increased after hypoxia induction (P < 0.001). VEGF121 overexpression significantly decreased LDH leakage rate (Figure 3A). Both NPS2390 and calpeptin had a synergistic effect with VEGF121 overexpression in decreasing LDH leakage rate, whereas the addition of CdCl2 did not have any additional effect (Figure 3A). A similar observation was made with caspase-3 activity (Figure 3B).

**Figure 3:** *VEGF can inhibit LDH leakage and Caspase-3 activity in hypoxia-treated cardiomyocytes. Experimental groups were - Group 1 (negative control group); Group 2 (hypoxia model group); Group 3 (hypoxia model group transfected with VEGF121); Group 4 (same as Group 3 but with calcium channel inhibitor CdCl2); Group 5 (same as Group 3 but with CaSR receptor inhibitor NPS2390); Group 6 (same as Group 3 but with calpain inhibitor Calpeptin). Shown are data for LDH leakage (A) and caspase-3 activity (B) from five independent replicates.* ***p <0.001 vs. control group; ^###^ *p<0.05 vs. Model group*; ^&^ *p<0.05 vs. Model + pcDNA3.1(+)/VEGF121 group*.

### Impact of hypoxia and VEGF on CaSR, Calpain, AIF, and tBid expression

Hypoxia significantly induced the expression of CaSR, calpain, AIF and tBid proteins (Figure 4; p<0.001). The expression levels of CaSR, calpain, AIF and tBid protein decreased after transfection of cardiomyocytes with pcDNA 3.1(+) / VEGF121, which was statistically significant when compared with the hypoxia model group (P<0.001). After cardiomyocyte treatment with NPS2390 and Calpeptin, the expression of tBid and AIF decreased, when compared to the Model + pcDNA 3.1(+) / VEGF121 group (P<0.05). After pcDNA3.1(+) / VEGF121 intervention in the cardiomyocytes, the expression of CaSR decreased, which was statistically significant when compared with the model group (p < 0.001). Again, a synergistic effect was observed with NPS2390 and calpeptin, but not with CdCl2.

**Figure 4:** *Impact of hypoxia, VEGF and indicated inhibitors on CaSR, Calpain, AIF, and tBid protein expression. Experimental groups were - Group 1 (negative control group); Group 2 (hypoxia model group); Group 3 (hypoxia model group transfected with VEGF121); Group 4 (same as Group 3 but with calcium channel inhibitor CdCl2); Group 5 (same as Group 3 but with CaSR receptor inhibitor NPS2390); Group 6 (same as Group 3 but with calpain inhibitor Calpeptin). (A, B) CaSR, Calpain, AIF and tBid were detected by Western blot. The Figure shows blots from 4 independent experiments. The graphs show densitometric analysis of blots from the four experimental replicates.* ***p <0.001 vs. control group; ^###^ *p<0.001 vs. Model group;* ^&^ p<0.05, ^&&^ p<0.01, ^&&&^ *p<0.001 vs. Model + pcDNA3.1(+) /VEGF121 group*.

## Discussion

The main mechanisms of cardiovascular disease caused by hypoxia-ischemia are myocardial apoptosis, fibroblast proliferation and vascular endothelial cell injury.^22^^–^^24^ VEGF, a specific mitogen of vascular endothelial cells, constitutes a family of secreted proteins, including VEGF121, VEGF165, VEGF189 and VEGF206, of which VEGF121 and VEGF165 are the main treatments for ischemic cardiomyopathy. It has been reported that VEGF121 can only bind to the VEGFR-2 receptor, and its VEGF-specific binding may attenuate angiogenesis, but other studies have also found that its ability to bind to heparin is weakened, which is more conducive to an anoxic environment. The direct injection of the VEGF121 plasmid into the pig myocardial infarction model has shown to improve blood supply and increase the number of collateral vessels at the same time, highlighting the potential of VEGF-based gene therapy. However, there are some limitations of instability, high price, and lack of high-dose application in clinical practice. At the same time, studies have also found that the gene product encoding secreted protein has a paracrine effect, compared with the cell's own protein.^25^

Cardiomyocytes are terminally differentiated cells, whose apoptosis is closely related to myocardial disease. Caspase-3 is the common mediator of apoptosis in cardiomyocytes.^26^^,^^27^ Heart failure shows different apoptotic rates of cardiomyocytes. In animal experiments of heart failure caused by hypoxia-reperfusion, the apoptotic rate of cardiomyocytes can reach 14%, and heart failure is caused by excessive pressure overload. The apoptotic rate of cardiomyocytes in the model is only 1%,^24^ which suggests that the study of apoptosis in a myocardial hypoxia model is of great value.

The immunoblot analysis confirmed a successful post-transfection VEGF121 expression in cardiomyocytes in the current study. Some studies have also confirmed that^9^ the quantitation of VEGF121 in the cell supernatant can confirm the secretion and expression of cardiomyocytes of an eukaryotic expression vector and has a strong proliferative effect on endothelial cells. LDH acts as an extra-leakage rate for intracellular enzymes, and its increase is a marker of cell damage. The LDH leak rate in the hypoxic model group was high and was rescued by VEGF121. Caspase-3 activation in hypoxia was inhibited by VEGF121 and the CaSR inhibitor NPS2390, suggesting that the calpain receptor and calpain are closely related to the mechanism of cardiomyocyte apoptosis.^10^ Given that our findings suggested that VEGF121 reverses apoptosis in cardiomyocytes post-hypoxia and CaSR inhibitor had a similar effect, it is suggested that VEGF121 is mediating its activity by inhibiting CaSR activation.

Because the dynamic changes in intracellular calcium levels are one of the important mechanisms of apoptosis,^28^^,^^29^ the intracellular pathways that affect calcium levels, mainly through Ca2+ channels and Na+/Ca2+ exchange, activate Ca2+ receptor pathways, such as CaSR. In other pathways,^30^ when hypoxia is stimulated, intracellular Ca2+ influx increases, CaN (Calcineurin) and Bid (Bcl-2 family protein) are activated, and the mitochondrial membrane permeability is significantly increased, thereby releasing Cyt-c to cytoplasmic activation of Caspase-3, thus promoting the damage of cardiomyocyte. Since Calpain is dependent on Ca2+ influx activation, activated Calpain can cleave and activate Bid. Due to its special BH3 domain, tBid can induce Bax/Bak translocation, resulting in increased mitochondrial membrane permeability, and tBid can also pass through it. The non-BH3 domain induces mitochondrial membrane structural changes, opens PTP, and activates calpain to degrade CaN inhibitory proteins, thereby activating CaN, which promotes phosphorylation of Bad, leading to apoptosis.^31^^–^^33^

Bid is the only Bcl-2 family that can be cleaved into small molecule Bids. After activation, it promotes the polymerization of Bak and Bax, or homopolymerizes itself, promotes the remodeling of mitochondrial inner membrane and the opening of mPTP pores, resulting in the release of Cyt-c.^34^^,^^35^ Our results showed that the expression of tBid protein in hypoxic cardiomyocytes was significantly increased. The level of tBid protein decreased with VEGF overexpression and further synergistically decreased with the addition of NPS2390 and calpeptin, but not with CdCl2 addition. It indicated that calcium ion and calpain caused a decrease in the activity of Bid after myocardial apoptosis caused by hypoxia. AIF is located in the mitochondrial inner membrane. When cardiomyocyte apoptosis occurs, the flavin protease containing NADH oxidase activity is released from the mitochondria into the cytoplasm and then translocated into the nucleus, involved in chromatin condensation and DNA fragmentation.^36^ In this experiment, CaSR receptor inhibitor (NPS2390) and calpain inhibitor (calpeptin) were added to induce a decrease in AIF protein levels, which was statistically significant. Our results suggested that VEGF121 is involved in cardiomyocyte apoptosis after AIF. Release was associated with CaSR activation, whereas the calpain antagonist calpeptin group and the pcDNA 3.1(+)/VEGF121 intervention group were not statistically significant, suggesting that it is not solely reliant on downstream calcium-dependent protease-induced apoptosis pathways. One potential limitation of our study is that the experiments were performed using neonatal cardiomyocytes; its relevance needs to be investigated in cardiomyocytes isolated from older rats and in *in vivo* pre-clinical models.

## Conclusion

It has been previously shown that VEGF can treat myocardial ischemia, increase vascular endothelial cell proliferation, increase myocardial collateral circulation and capillary density, thereby improving myocardial blood supply in the ischemic area and improving cardiac function.^37^^–^^39^ However, damage to vascular endothelial cells, cardiomyocyte hypertrophy, cardiomyocyte apoptosis and fibroblast proliferation are also key pathophysiological changes in ischemic cardiomyopathy. In this study, based on the intervention groups, VEGF121 affected cardiomyocyte proliferation, confirming the biological activity of eukaryotic plasmids on cardiomyocytes. In conclusion, our findings provided a framework for future VEGF-based gene therapy approaches in cardiovascular disorders.
